# Genomic and Functional Analysis of a Novel Yeast *Cyberlindnera fabianii* TBRC 4498 for High-Yield Xylitol Production

**DOI:** 10.3390/jof11060453

**Published:** 2025-06-13

**Authors:** Pawarin Bonthong, Benjarat Bunterngsook, Wuttichai Mhuantong, Katesuda Aiewviriyasakul, Wipawee Sritusnee, Verawat Champreda, Hataikarn Lekakarn

**Affiliations:** 1 Department of Biotechnology, Faculty of Science and Technology, Rangsit Campus, Thammasat University, Pathum Thani 12120, Thailand; pawarinbonthong@gmail.com; 2 Enzyme Technology Research Team, Biorefinery Technology and Bioproduct Research Group, National Center for Genetic Engineering and Biotechnology, 113 Thailand Science Park, Khlong Luang, Pathum Thani 12120, Thailand; benjarat.bun@biotec.or.th (B.B.); wuttichai.mhu@biotec.or.th (W.M.); katesuda.aie@biotec.or.th (K.A.); w.sritusnee@gmail.com (W.S.); verawat@biotec.or.th (V.C.)

**Keywords:** *Cyberlindnera fabianii*, xylitol, xylose, cell factory, genome

## Abstract

The development of yeast cell factories for efficient xylose utilization and xylitol production is crucial for advancing sustainable biotechnological processes. Xylose, a major component of lignocellulosic biomass, presents challenges for microbial conversion due to its complex metabolic pathways. This study presents the genomic perspective and xylitol production capability of a novel xylose utilizing yeast *Cyberlindnera fabianii* TBRC 4498. Genome sequencing and functional annotation revealed key metabolic networks and genes involved in the xylose metabolism pathway, providing insights into the strain’s performance. The *Cy. fabianii* TBRC 4498 had excellent growth and xylose assimilation at a broad range of xylose concentrations from 40 to 140 g/L, with the highest growth rate at 80 g/L of xylose. The highest xylitol production yield (83.19 g/L) was detected from 120 g/L of xylose at 30 °C for 72 h, equivalent to 0.65 g xylitol/g xylose and 1.16 g/L/h productivity. Remarkably, *Cy. fabianii* TBRC 4498 produced high-purity xylitol, achieving over 95% homogeneity without forming undesirable byproducts, such as acid or ethanol. These results demonstrated the potential of *Cy. fabianii* TBRC 4498 as a whole-cell biocatalyst for xylitol production using high xylose concentrations, offering a promising microbial cell factory for large-scale xylitol production from lignocellulosic sugar.

## 1. Introduction

Xylitol, a five-carbon sugar alcohol (C_5_H_10_O_5_), is widely recognized as a low-calorie sweetener used in the food, pharmaceutical, and dental care industries. The market value of xylitol was USD 713.4 million in 2023 and is projected to reach USD 1086.8 million by 2033, reflecting a compound annual growth rate (CAGR) of 4.3%. Xylitol exhibits distinct characteristics, including a sweetness comparable to sucrose and a caloric value of 2.4 kcal/g, which is 40% lower than sucrose [1,2]. Xylitol is metabolized via insulin-independent pathways and has a negligible impact on blood glucose levels, making it a suitable alternative sweetener for individuals with diabetes [3]. Xylitol inhibits oral bacterial growth, thereby reducing the risk of tooth decay [4]. Traditionally, xylitol production has depended on the chemical hydrogenation of xylose using Raney nickel catalysts under high pressure and temperature, resulting in high waste management costs and energy consumption [5]. To address the limitations of the chemical process, microbial fermentation using yeast whole-cell biocatalysis has gained increasing attention as an environmentally friendly and cost-effective alternative. Yeasts can convert xylose into xylitol through mild conditions of enzymatic pathways, offering a promising biotechnological approach for sustainable production.

Several yeast species are capable of producing xylitol with varying yields. *Candida tropicalis*, classified as a risk group 2 organism, has been identified as an effective xylitol producer, with certain strains, such as *C. tropicalis* JA2, exhibiting high conversion efficiency from xylose, producing up to 109.5 g/L of xylitol [6]. In contrast, yeasts classified as risk group 1, such as *Wickerhamomyces rabaulensis*, *Wickerhamomyces anomalus*, *Cyberlindnera xylosilytica*, *Cyberlindnera galapagoensis*, *Cyberlindnera dasilvae*, and *Meyerozyma guilliermondii*, produce lower concentrations of xylitol, typically ranging from 7.25 to 46.87 g/L, using xylose concentrations between 40 and 60.65 g/L [7,8,9,10]. Due to the regulatory requirements associated with handling risk group 2 yeasts, which can increase operation costs, there is growing interest in utilizing risk group 1 yeasts capable of tolerating high xylose concentrations and producing high amounts of xylitol within a short fermentation period as a cost-effective alternative for industrial applications. Therefore, this study aims to identify a novel yeast with this capability. Additionally, understanding the metabolic pathways, transporters, and genes involved in xylitol biosynthesis is crucial for optimizing the conditions necessary for using newly discovered yeasts.

*Cyberlindnera fabianii*, previously known as *Lindnera fabianii*, *Hansenula fabianii*, and *Pichia fabianii*, has demonstrated notable potential in various biotechnological applications. Regarding biosafety classification, *Cy. fabianii* was classified as a risk group 1 microorganism. This yeast is particularly valuable in food fermentation, contributing to the creation of complex aroma profiles and ester production, which is essential for the synthesis of various ester substances in alcoholic beverages [11,12,13,14]. Additionally, *Cy. fabianii* has proven effective in dye mycoremediation, utilizing oxidase and reductase enzymes to biodegrade azo dyes [15]. These characteristics make it a promising candidate for biotechnology applications. However, to fully realize its potential, further research is needed to better understand its metabolic processes, regulatory networks, and transport mechanisms, as well as to develop advanced genetic manipulation systems.

This study introduces the xylitol-producing capability of a novel *Cyberlindnera* species, *Cy. fabianii* TBRC 4498, and provides a genomic analysis of the metabolic pathways involved in xylitol production. Notably, *Cy. fabianii* TBRC 4498 produces high yields of xylitol under aerobic conditions across a wide range of xylose concentrations (40–140 g/L). This demonstrates its potential as a promising xylitol producer with the ability to tolerate high osmotic stress, making it suitable for xylitol production from lignocellulosic sugars. Furthermore, this process offers a sustainable and environmentally friendly approach to xylitol production.

## 2. Materials and Methods

### 2.1. Strain, Chemicals, and Culture Media

A xylitol-producing yeast, *Cy. fabianii* TBRC 4498, isolated from mangrove forest water, was obtained from the Thailand Bioresource Research Center (TBRC). For cell preservation, the yeast strain was grown in YPD medium (10 g/L of yeast extract, 20 g/L of peptone, and 20 g/L of dextrose). During the fermentation step for xylitol production, the yeast strain was cultured with YPX medium containing 10 g/L of yeast extract, 0.2 g/L of MgSO_4_·7H_2_O, 5 g/L of KH_2_PO_4_, and 1 g/L of urea with varying xylose concentrations (40–140 g/L) as a carbon source. All chemicals and standards were purchased from the companies Sigma-Aldrich (St. Louis, MO, USA), Carlo Erba (Milan, Italy), Difo (Detroit, MI, USA), Fluka (Buchs, Switzerland), and Gibco (Grand Island, NY, USA).

### 2.2. Genome Sequence Analysis

Genomic DNA from *Cy. fabianii* TBRC 4498 was extracted using the GeneJET Genomic DNA Purification Kit (Thermo Fisher Scientific, Waltham, MA, USA), and the concentration of the extracted genomic DNA was quantified using the NonoDrop^TM^ 2000 spectrophotometer (Thermo Fisher Scientific, Waltham, MA, USA). The genome sequencing of *Cy. fabianii* TBRC 4498 was performed through the Illumina NovaSeq 6000 platform (Novogene, Singapore) using 2 × 250 bp paired-end reads. Raw paired-end sequences were initially used to perform the sequence quality control by removing low-quality sequences (average Phred Quality score < 30) and trimming barcode and adapter sequences using FASTP (version 0.20.1) [16]. Cleaned sequences were then used to perform the de novo genome assembly using SPAdes (version 3.15.4) [17] with a minimum contiguous sequence (contig) length of 500 bps. To enhance the contiguity of the assembled genome, scaffolding was performed by RagTag (version 2.1.0) [18] using the reference genome of *Cy. fabianii* JOY008 (GCA_022641835.1). The quality of the assembled genome was assessed using QUAST (version 5.2.0) for contiguity metrics [19] and BUSCO (version 5.4.2) [20] for completeness of genes based on conserved single-copy orthologs. Gene prediction was conducted using AUGUSTUS (version 3.4.0) [21], incorporating training protein data from *Cy. fabianii* 65 (GCA_001983305.1) and four pre-trained *Saccharomycetes* yeast models provided in AUGUSTUS. Predicted gene models were then weighted and merged into consensus gene structures using EVidenceModeler (EVM) version 1.1.1 [22].

Functional annotation of predicted proteins was performed using BLASTP (version 2.12.0+) searches against the UniProt fungal protein database [23], retrieved in November 2024. All potential carbohydrate-active enzymes were also predicted using dbCAN2, a web server for automated carbohydrate-active enzyme annotation [24]. Functional pathway annotations were mapped via GhostKOALA (version 3.1) [25]. The Gene Ontology (GO) annotations of all predicted genes were assigned using PANNZER2 (version 15.6.2023) in order to describe the molecular function, cellular component, and biological process of genes in the genome [26]. Clusters of Orthologous Genes (COGs) were annotated toward EggNOG v5.0 [27]. For protein structure annotation, the conserved domain was analyzed by the NCBI Conserved Domains Database (CDD) [28]. The signal peptide involved in the protein secretion pathway was analyzed by SignalP 6.0 [29].

### 2.3. Taxonomic Classification

The taxonomic classification of *Cy. fabianii* TBRC 4498 was determined using a combination of molecular markers and genome-based comparison approaches. First, the internal transcribed spacer (ITS) region of the assembled genome was identified using ITSx [30]. Then, the taxonomy was inferred by homology search against the NCBI ITS reference database using BLASTN. Genomes with high ITS sequence similarity to *Cyberlindnera* species were subsequently subjected to genome-wide comparison by calculating the average nucleotide identity (ANI) using FastANI [31] to evaluate genetic relatedness among publicly available *Cyberlindnera* genomes. In addition, phylogenetic inference was conducted using 61 universally conserved fungal marker genes obtained from the Universal Fungal Core Genes (UFCG) database [32], providing high-resolution insight into evolutionary relationships.

### 2.4. Xylitol Production

The *Cy. fabianii* TBRC 4498 yeast strain was refreshed on YPX agar containing 20 g/L of xylose and incubated at 30 °C for 3 days. For starter preparation, the half-loop of the colony was cultured in 50 mL of fresh YPX medium containing 20 g/L of xylose in a 250 mL baffled flask at 30 °C with a 200 rpm shaking speed for 24 h. For the xylitol production step, the starter culture was inoculated into 50 mL of YPX medium containing four different xylose concentrations: 40, 80, 120, and 140 g/L, in 250 mL baffled flasks, with a final cell concentration of 0.05 optical density at 600 nm (OD_600_). The cell culture was incubated at 30 °C with a 200 rpm shaking speed. Cell growth and xylitol production performance were monitored by sample withdrawing every 24 h until 168 h. For growth measurement, OD_600_ values were measured by a NanoDrop™ One/One^C^ Microvolume UV-Vis Spectrophotometer (Thermo Scientific, MA, USA) and properly diluted. Afterward, the yeast cell dry weight (g/L) was determined. The experiment was performed in triplicate and the data have been expressed as mean ± standard error.

### 2.5. Product Profile Analysis Using HPLC

In order to measure the concentration of xylose, xylitol, and other intermediates produced in cell culture, including ethanol and organic acids, 1 mL of the collected sample at 0, 24, 48, 72, 96, 120, 144, and 168 h was separated by centrifugation at 25 °C and 12,000 rpm for 5 min. The transparent supernatant was filtered through a 0.22 μm nylon syringe filter. Then, the filtrate was subjected to the Dionex UltiMate 3000 High-Performance Liquid Chromatography (HPLC) system (Thermo Scientific, MA, USA) equipped with an Aminex HPX-87H column (BIO-RAD, Hercules, CA, USA) and connected through a Refractive Index (RI) detector (Shodex, Tokyo, Japan). The column was maintained at 65 °C using 5 mM H_2_SO_4_ as the mobile phase at a flow rate of 0.5 mL/min. The various xylose and xylitol concentrations of 2, 4, 6, 8, and 10 g/L were used as standards for product qualification and quantification measurements.

### 2.6. Cell Growth Comparison in Different Xylose Concentrations

The growth rate of *Cy. fabianii* TBRC 4498 in various xylose concentrations was evaluated on YPX agar plates with four different xylose concentrations of 40, 80, 120, and 140 g/L. Firstly, the yeast cell was refreshed on a YPX agar plate containing 20 g/L of xylose concentration from the glycerol stock and incubated at 30 °C for 72 h. A colony from the cell plate was subsequently selected by an inoculating loop into 5 mL of YPX medium containing 20 g/L of xylose and incubated at 30 °C with a 200 rpm shaking speed for 72 h. Then, the cell culture was diluted to final absorbance at 600 nm (OD_600_) of 0.1 and subsequently diluted with serial dilution from 10^−2^ to 10^−5^ using YPX medium with 20 g/L of xylose. A 20 µL cell suspension with different dilutions was spotted on dried-surface YPX agar plates. The plates were dried at room temperature for 20 min and subsequently incubated at 30 °C for 72 h.

## 3. Results and Discussion

### 3.1. Sugar-Utilizing Profile of Cy. fabianii TBRC 4498

**Figure 1:**
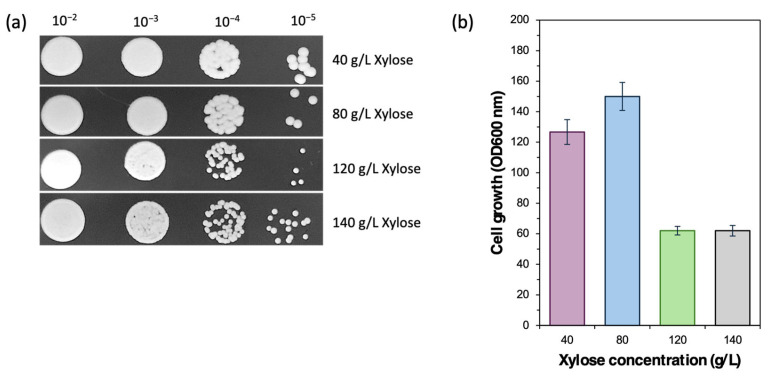
Growth ability of *Cy. fabianii* TBRC 4498 using xylose as the carbon source. The yeast strain was grown in YPX agar supplemented with 40, 80, 120, and 140 g/L of xylose. (**a**) Spot assay. An equal number of cells were spotted at 10^−2^ and three serial dilutions of 10^−3^, 10^−4^, and 10^−5^ (left to right) on YPX agar and incubated at 30 °C for 72 h. (**b**) Cell growth measured by absorbance at 600 nm.

*Cy. fabianii* is a species of non-*Saccharomyces* yeast that has undergone several taxonomic reclassifications, previously known as *L. fabianii*, *H. fabianii*, and *P. fabianii* [33,34]. In particular, *Cy. fabianii* exhibited especially promising features due to its metabolic versatility and sensory contributions in fermentation. Notably, it enhances flavor complexity and generates fruity, ester-rich aroma profiles, making it a promising alternative to *Saccharomyces* species in brewing and other fermented food applications [13,35,36]. In this study, the wild strain *Cy. fabianii* TBRC 4498 was isolated from water in a mangrove forest in Thailand. Cell visualization using confocal microscopy revealed that *Cy. fabianii* TBRC 4498 cells had a diameter of 2–4 µm. In sugar assimilation screening, *Cy. fabianii* TBRC 4498 completely consumed 80 g/L of glucose and fructose within 24 h, and 80 g/L of xylose within 48 h. These results demonstrate that *Cy. fabianii* TBRC 4498 can efficiently utilize both C6 and C5 sugars, indicating its potential for producing functional biochemicals from lignocellulosic sugars. Focusing on xylose, a typically underutilized lignocellulosic sugar, *Cy. fabianii* TBRC 4498 was able to assimilate a wide range of xylose concentrations, from 40 to 140 g/L (Figure 1), further highlighting its promise as a natural xylose-utilizing yeast strain for biotechnological applications.

### 3.2. Genome Sequence and Functional Annotation

**Figure 2:**
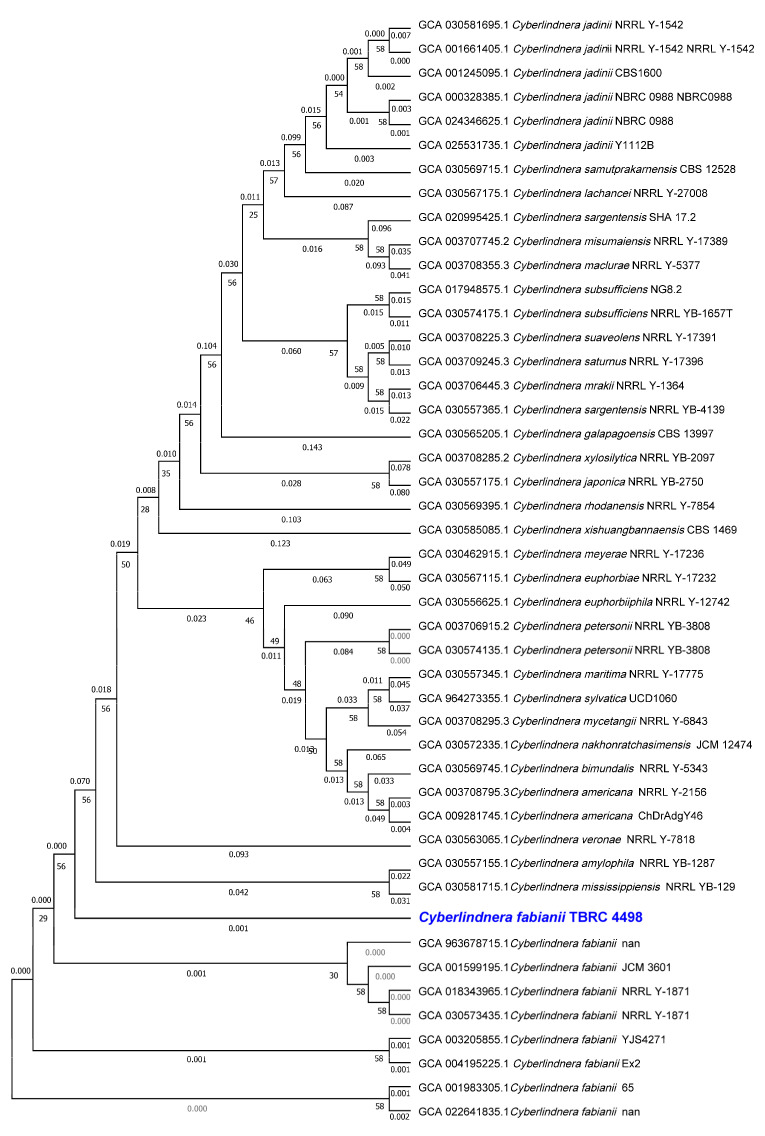
The phylogenetic tree illustrates the evolutionary relationships between *Cy. fabianii* TBRC 4498 and 45 closely related species within the genus *Cyberlindnera*. The phylogeny was inferred using a concatenated set of 61 conserved marker genes identified from the genome of *Cy. fabianii* TBRC 4498 and the genomes of related species.

To further explore the underlying mechanisms of xylose utilization and other functional capabilities, the draft genome of *Cy. fabianii* TBRC 4498 was sequenced and annotated. Genome sequencing was performed using the Illumina 150 PE platform, resulting in a final assembly of 77 contigs, with the longest contig length of 1,092,463 bp. The genome size of *Cy. fabianii* TBRC 4498 was 12,320,282 bp, with an average G + C content of 43%. This Whole Genome Shotgun project has been deposited at DDBJ/ENA/GenBank under the accession number JBNQWT000000000. Genome sequence comparison and evolutionary relationship analysis revealed that the genome of *Cy. fabianii* TBRC 4498 was closely related to several *Cyberlindnera* species, including *Cy. fabianii*, *Cy. jadinii*, *Cy. samutprakarnensis*, and *Cy. lachancei*. Particularly, it was closely related to *Cy. jadinii*, a yeast species known for its ability to metabolize xylose and produce sugar alcohols [37]. Notably, the *Cy. fabianii* TBRC 4498 genome exhibited the highest similarity with *Cy. fabianii*, as reflected by a short genetic distance (Figure 2). This close genetic relationship suggests a strong phylogenetic connection between the two strains, which may indicate shared metabolic pathways and functional traits. Based on the whole-genome comparison, *Cy. fabianii* TBRC 4498 was closely related to the *Cy. fabianii* YJS4271 genome with approximately 12.3 Mb (44.4% G + C content) and contained 5944 putative protein-coding genes [38], consistent with the *Cy. fabianii* 65 genome containing approximately 12.3 Mb (44.4 G + C content) with 5509 putative genes [14].

**Figure 3:**
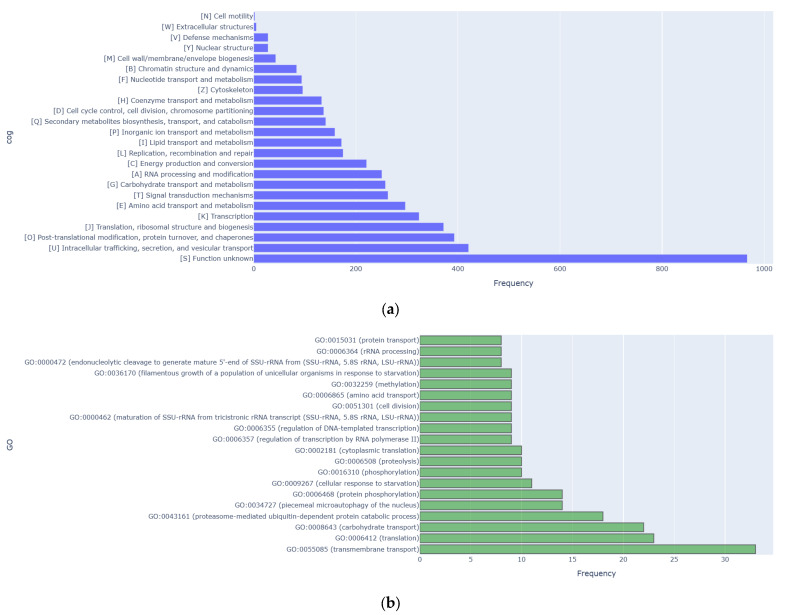
Functional annotation of putative proteins found in the *Cy. fabianii* TBRC 4498 genome. (**a**) Clusters of Orthologous Genes (COGs) classification. (**b**) Gene Ontology (GO) classification.

Regarding protein function annotation based on Clusters of Orthologous Genes (COGs), the genome of *Cy. fabianii* TBRC 4498 contained 2630 putative protein-coding genes, in which 967 were identified as hypothetical proteins (Figure 3a). Functional classification by COGs revealed that *Cy. fabianii* TBRC 4498 contained a high proportion of proteins involved in intracellular trafficking (COG U), posttranslational modification (COG O), and translational (COG J), as well as carbohydrate transport and metabolism (COG G). Based on functional annotation toward the Gene Ontology (GO) database, a total of 414 genes were identified in the genome of *Cy. fabianii* TBRC 4498. The largest group of annotated genes was associated with transmembrane transport (GO: 0055085; 33 genes), followed by genes related to translation (GO: 0006412; 23 genes) and carbohydrate transport (GO: 0008643; 22 genes; Figure 3b).

**Figure 4:**
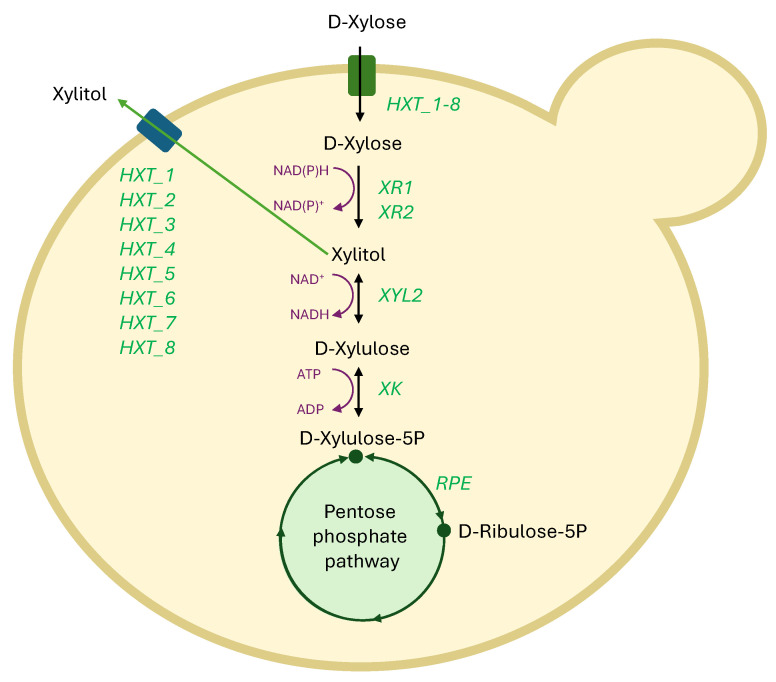
The proposed metabolic pathway involved in xylose assimilation and xylitol biosynthesis in *Cy. fabianii* TBRC 4498. The putative genes in the metabolic pathway include fungal hexose transporter (HXT), NADPH-dependent D-xylose reductase (XR), xylitol dehydrogenase (XYL2), xylulose kinase (XK), and D-ribulose-5-phosphate 3-epimerase (RPE).

According to functional gene annotation based on the UniProt and KEGG databases, the proposed metabolic pathway involved in xylose utilization in *Cy. fabianii* TBRC 4498 comprised eight putative fungal hexose transporter-encoding genes (HXT_1–8; Query Gene IDs: CHR1.785, CHR1.835, CHR1.967, CHR1.1111, CHR4.785, CHR5.787, CHR8.33, and CHR9.40) with E-values of 7.38 × 10^−97^ to 1.26 × 10^−143^, indicating high sequence similarity. These genes also showed moderate similarity to D-xylose transporter (XylE), a xylose/proton symporter, with E-values of 1.03 × 10^−42^ to 3.59 × 10^−66^. Overall, these transporters exhibited the highest sequence similarity to members of the HXT subfamily, suggesting a potential role in hexose and xylose transport across the plasma membrane, particularly in the absence of more specific xylose transporters (Figure 4). Basically, the fungal hexose transporter (HXT) subfamily comprises functionally redundant proteins that primarily mediate the facilitated diffusion of glucose and other hexoses, such as galactose and fructose, across the plasma membrane. Several HXT transporters also contribute to xylose uptake, although they typically exhibit higher affinity for glucose than xylose. In contrast, xylose-specific transporters function through a proton symport mechanism, enabling the active assimilation of xylose, even at low extracellular concentrations. This transport strategy is generally more efficient for xylose uptake and is commonly found in native xylose-fermenting yeasts, such as *Kluyveromyces marxianus*, *Candida intermedia*, *Scheffersomyces stipitis*, and *Candida sojae* [39,40,41]. Interestingly, the *Cy. fabianii* TBRC 4498 genome contained two NADPH-dependent D-xylose reductase (XR1 and XR2) genes that represent key enzymes for xylitol biosynthesis (Query Gene IDs: CHR9.18 and CHR9.222). Xylitol accumulation within yeast cells typically serves as an osmolyte that can protect yeast cell water hydration, increasing the osmotic pressure and retaining water within the cell, particularly under stressful conditions [42]. However, xylitol accumulation is influenced by metabolic capacity and its tolerance to the sugar alcohol. Importantly, excessive intracellular accumulation of xylitol can lead to cellular toxicity, primarily due to osmotic stress, which disturbs cellular function and integrity. To mitigate this stress, xylitol is actively transported from the intracellular space to the extracellular environment through specific membrane-bound transporters. In *Cy. fabianii* TBRC 4498, xylitol is hypothesized to be exported predominantly via eight fungal HXT-like proteins (HXTs). Additionally, the remaining xylitol within the cell is further metabolized through the pentose phosphate pathway (PPP). Xylitol is first oxidized to D-xylulose by xylitol dehydrogenase (XYL2; Query Gene ID: CHR1.922). Subsequently, D-xylulose is phosphorylated by xylulose kinase (XK), encoded by the gene CHR4.52, converting it to D-xylulose-5-phosphate (D-Xylulose-5P). This phosphorylated intermediate is further processed by D-ribulose-5-phosphate 3-epimerase (RPE; encoded by Query Gene ID: CHR3.267), which catalyzes the conversion of D-xylulose-5P into D-ribulose-5-phosphate (D-ribulose-5P). D-ribulose-5P serves as a key intermediate in the pentose phosphate pathway, which is crucial for cellular biosynthesis and maintaining cellular redox balance. In addition, D-xylulose-5-phosphate also acts as a key intermediate linking xylose metabolism to the glycolysis pathway, being enzymatically converted to fructose-6-phosphate and glyceraldehyde-3-phosphate by putative transketolases (Query Gene IDs: CHR3.388 and CHR9.17). Glyceraldehyde-3-phosphate is further metabolized to pyruvate, which enters the TCA cycle and contributes to ATP synthesis through oxidative phosphorylation [43,44]. The genomic analysis of *Cy. fabianii* TBRC 4498 revealed key metabolic pathways and sugar transport genes involved in xylose assimilation, supporting efficient utilization and minimal toxic byproduct accumulation, thereby supporting enhanced xylitol production. These findings demonstrated the basis for further genetic and metabolic engineering to optimize the performance of *Cy. fabianii* TBRC 4498 in industrial fermentation processes.

### 3.3. Xylitol Production Capability of Cy. fabianii TBRC 4498

**Figure 5:**
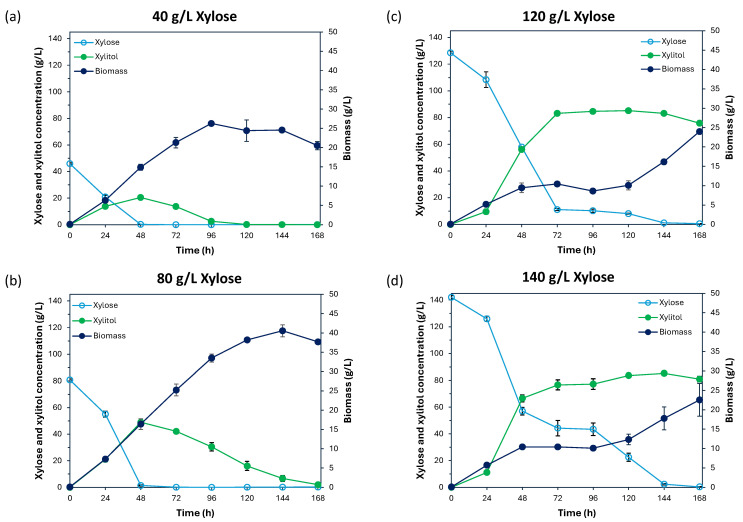
Xylitol production and growth rates of the *Cy. fabianii* TBRC 4498 yeast strain were assessed using xylose as a substrate. The yeast strain was cultivated in four different concentrations of xylose, including (**a**) 40 g/L, (**b**) 80 g/L, (**c**) 120 g/L, and (**d**) 140 g/L at 30 °C and 200 rpm for 24–168 h. All experimental results are based on data obtained from three independent replicates.

Subsequently, xylose utilization efficiency and xylitol production were investigated under varying xylose concentrations in YPX medium, with four different conditions: 40, 80, 120, and 140 g/L of xylose. Regarding cultivation in YPX medium with 40 g/L of xylose (actual xylose concentration measured by HPLC was 45.95 g/L), *Cy. fabianii* TBRC 4498 efficiently consumed 45.72 g/L of xylose within 48 h, leaving only 0.23 g/L, indicating nearly 100% xylose consumption (Figure 5a). The yeast showed rapid growth between 24 and 96 h, with a dry cell weight of 6.31–26.25 g/L, followed by a stable growth pattern at 120–144 h. At 48 h, the yeast produced the highest xylitol concentration (20.49 g/L), with conversion rates of 44.82% based on xylose consumption and 44.60% based on the initial xylose concentration, with a low ethanol concentration for 1.73 g/L. The xylitol productivity was 0.43 g/L/h, and the yield was 0.45 g of xylitol/g of xylose. While xylitol productivity was highest at 24 h (0.57 g/L/h), the 48 h fermentation yielded the most efficient overall xylitol production. In YPX medium with 80 g/L of xylose, *Cy. fabianii* TBRC 4498 exhibited the highest growth, achieving 40.56 g/L dry cell weight at 144 h with nearly complete xylose consumption within 48 h (Figure 5b). Starting with 80.84 g/L of xylose, only 1.40 g/L remained, resulting in a 98.27% xylose utilization rate. During this period, the yeast produced the highest xylitol concentration (48.97 g/L), with conversion rates of 61.67% from consumed xylose and 60.59% from the initial xylose concentration. The ethanol production was detected as a minor product for this stage (6.04 g/L). The xylitol productivity was 1.02 g/L/h, and the yield was 0.61 g of xylitol/g of xylose, demonstrating the yeast’s high efficiency in xylitol production over a short period. In the cultivation of *Cy. fabianii* TBRC 4498 with 120 g/L of xylose (Figure 5c), the yeast nearly completely consumed the xylose by 144 h. At 72 h, the yeast produced 83.19 g/L of xylitol, representing the highest xylitol productivity at 1.16 g/L/h. At this point, the xylitol conversion rate from the starting xylose was 64.72%, with a yield of 0.65 g of xylitol/g of xylose and only 4.01 g/L of ethanol concentration. Notably, after 72 h, the yeast maintained a high xylitol concentration throughout the remainder of the fermentation, unlike the previous conditions with lower xylose concentrations, where xylitol production declined. This highlights the efficiency of *Cy. fabianii* TBRC 4498 in xylitol production under high xylose concentrations. At 120 g/L of xylose, growth was steady from 24 to 120 h, reaching a peak at 168 h (23.98 g/L dry cell weight). Finally, in YPX medium with 140 g/L of xylose, the highest concentration tested in this study (Figure 5d), *Cy. fabianii* TBRC 4498 consumed nearly all the xylose by 144 h. At 48 h, the yeast achieved the highest xylitol productivity in this experiment, 1.39 g/L/h, demonstrating efficient xylose-to-xylitol conversion in a short time under high xylose conditions. The maximum xylitol production reached 85.28 g/L, with conversion rates of 60.02% from the starting xylose and 61.01% from the consumed xylose. A low ethanol byproduct concentration of 2.30 g/L was also observed. At 144 h, the xylitol productivity decreased to 0.59 g/L/h. While the total xylitol production was high, the slower rate of production over 144 h resulted in lower productivity. Nonetheless, the yeast maintained a high xylitol concentration throughout the fermentation process. At the highest xylose concentration of 140 g/L, growth was similar to that in 120 g/L of xylose, with stable growth from 24 to 120 h, and the highest growth at 22.55 g/L dry cell weight at the end of fermentation. Remarkably, *Cy. fabianii* TBRC 4498 demonstrated unique performance in producing high-purity xylitol at respective conditions, achieving over 95% homogeneity without the formation of undesirable byproducts, such as organic acids and ethanol, distinguishing *Cy. fabianii* TBRC 4498 as a superior microbial candidate for industrial-scale xylitol production. This purity is critical for applications in food, pharmaceuticals, and other industries, highlighting its potential as a robust and efficient platform for sustainable and high-quality xylitol biosynthesis.

Regarding xylitol production efficiency, *Cy. fabianii* TBRC 4498 exhibited effective xylitol yields under different conditions, with the highest xylitol productivity (1.16 g/L/h) at 120 g/L of xylose for 72 h, suggesting this time point as optimal for cost-effective production (Table 1). The comparison of *Cy. fabianii* TBRC 4498 with other yeast strains demonstrated its high efficacy in xylitol production. The well-established xylose-fermenting yeast, *Cy. xylosilytica* UFMG-CM-Y309, exhibited notable conversion efficiency, with a xylitol yield of 0.77 g/g of starting xylose and 0.87 g/g of consumed xylose; however, it started from a low xylose concentration of 60.65 g/L [9]. In contrast, *W. anomalus* 740 and *W. rabaulensis* UFMG-CM-Y3716 showed xylitol yields of 0.62 and 0.65 g/g of starting xylose, respectively [8,9]. For *Candida* species, the *C. tropicalis* JH030 and *Candida guilliermondii* FTI20037 produced 31.1 g/L of xylitol (0.68 g/g of starting xylose) and 38.4 g/L of xylitol (0.67 g/g of starting xylose) from 45.8 and 57.7 g/L of xylose, respectively [45,46]. The *C. tropicalis* As 2.1776 exhibited a strong xylitol production capacity, with a xylitol yield of 58.3 g/L (equivalent to 0.72 g/g of xylose and 0.61 g/L/h) from 80 g/L of starting xylose in corncob hydrolysate medium at 96 h. Moreover, *C. tropicalis* As 2.1776 exhibited an osmotolerant in 160 g/L of xylose; however, it showed lower xylitol productivity with 0.32 g/L/h.

The *Cy. fabianii* TBRC 4498, a xylose-fermenting yeast, exhibited notable robustness under high xylose concentrations and osmotic pressure, with high xylitol productivity observed across a wide xylose range (40–140 g/L). This performance may be attributed to the presence of multiple NADPH-dependent D-xylose reductase genes (XR1 and XR2), which facilitate the reduction of xylose to xylitol. High xylose concentrations can pose metabolic stress; however, gene redundancy in xylose reductases may enable more efficient flux through the pathway, minimizing the accumulation of toxic intermediates and enhancing xylose tolerance. Two isoforms of xylose reductase—an NADPH-specific XR (msXR) and a dual-specificity NADPH/NADH XR (dsXR)—have been identified in the xylose-fermenting yeast *C. intermedia*, which exhibited high efficiency in ethanol production from xylose [47]. Additionally, the *Cy. fabianii* TBRC 4498 genome encoded eight putative hexose transporters (HXT_1–8), which are expected to contribute to the regulation of xylose uptake and xylitol export. An efficient transport system helps mitigate intracellular xylose toxicity, supporting improved metabolic stability under stress conditions. Regarding the genome perspective, the XR/XDH pathway was the primary route for xylose assimilation in *Cy. fabianii* TBRC 4498. XR1 and XR2 preferred NADPH, while xylitol dehydrogenase (XYL2) used NAD⁺, leading to a redox imbalance during xylose metabolism, resulting in excess NADH accumulation. The *Cy. fabianii* TBRC 4498 genome encoded multiple NAD(P)H-dependent oxidoreductases that regenerated cofactors and maintained redox balance, thereby supporting oxygen-independent xylose utilization, as observed in *S. stipitis* [48,49]. Regarding these results, in conclusion, *Cy. fabianii* TBRC 4498 represents a novel effective yeast strain for xylitol production at a broad xylose concentration (40–140 g/L), balancing both yield and productivity, with distinct advantages in time-efficient fermentation and minimizing product loss due to further metabolism. The high performance observed may be attributed to the presence of multiple NADPH-dependent D-xylose reductase genes (XR1 and XR2), which enhanced xylose reduction and minimized toxic intermediate accumulation. Gene redundancy likely improved metabolic flux and tolerance to high xylose concentrations. Similar to *C. intermedia*, which harbors both NADPH-specific and dual-specificity xylose reductases, *Cy. fabianii* TBRC 4498 possessed diverse xylose-assimilating enzymes. Its genome also encoded eight putative hexose transporters (HXT_1–8), potentially regulating xylose uptake and xylitol export to alleviate intracellular stress. Furthermore, the XR/XDH pathway, characterized by cofactor imbalance (NADPH for XR and NAD⁺ for XDH), was supported by multiple NAD(P)H-dependent oxidoreductases that helped maintain redox balance and enabled oxygen-independent xylose metabolism, as seen in *S. stipitis*.

**Table 1:** Comparing xylitol production with previous reports.

Yeast Strain	Xylose (g/L)	Xylitol (g/L)	Yield (g/g) (Based on Starting Xylose)	Yield (g/g) (Based on Consumed Xylose)	Productivity (g/L/h)	Conversion (%) (Based on Starting Xylose)	Time (h)	Risk Group	Reference
*Cyberlindnera fabianii* TBRC 4498	128.54	83.19	0.65	0.71	1.16	64.72	72	1	This work
*Cyberlindnera (Williopsis) saturnus*	150	38.63	0.26	0.54	0.27	25.75	144	1	[50]
*Cyberlindnera galapagoensis* sp. nov. CLQCA-24SC-025	50	17.01	0.34	0.50	0.23	34.02	72	n.r.	[7]
*Cyberlindnera xylosilytica* UFMG-CM-Y309	60.65	46.87	0.77	0.87	0.65	77.27	72	n.r.	[9]
*Meyerozyma guilliermondii* B12	40	5.00	0.13	0.33	0.11	12.50	44	2	[8]
*Meyerozyma guilliermondii* IM/UFRJ	109	40.8	0.37	0.71	0.85	37.43	48	2	[51]
*Wickerhamomyces anomalus* 740	40	24.75	0.62	0.75	0.56	61.88	44	1	[8]
*Wickerhamomyces rabaulensis* UFMG-CM-Y3716	60.65	39.66	0.65	0.69	0.55	65.39	72	n.r.	[9]
*Candida tropicalis* CLQCA-24F-125	50	25.63	0.51	0.67	0.36	51.26	72	2	[7]
*Candida xylopsoci* SL6	50	5.00	0.10	n.r.	0.07	10.00	72	n.r.	[10]
*Candida tropicalis* isolate Balki1	20	4.25	0.21	n.r.	0.06	21.25	72	2	[52]
*Candida guilliermondii* FTI20037	57.7	38.4	0.67	0.73	1.92	66.55	20	2	[46]
*Candida tropicalis* As 2.1776	80	58.3	0.72	0.74	0.61	72.87	96	2	[53]
*Candida tropicalis* JH030	45.8	31.1	0.68	0.71	0.44	67.90	70	2	[45]
*Pichia kudriavzevii* R5	50	5.50	0.11	n.r.	0.08	11.00	72	2	[10]
*Pichia stiptis* CBS 5773	52	31.68	0.61	0.96	0.44	60.92	72	1	[54]
*Kluyveromyces marxianus* CCA510	36.44	12.27	0.35	0.38	0.106	34.93	120	1	[55]

n.r. = not reported.

## 4. Conclusions

This work demonstrated the efficiency of a novel yeast, *Cy. fabianii* TBRC 4498, on high xylitol production in a wide xylose range (40–140 g/L), with the highest productivity of 1.16 g/L/h at 128 g/L of xylose. Moreover, the genome perspective of *Cy. fabianii* TBRC 4498 revealed key metabolic networks and genes involved in the xylose metabolism pathway, providing insights into the strain performance. The development of *Cy. fabianii* TBRC 4498 as a novel yeast cell factory for xylitol production offers a promising strategy for sustainable biomanufacturing from lignocellulosic biomass.

## Data Availability

The raw data supporting the conclusions of this article will be made available by the authors upon request.
